# Protection of metabolic-haemodynamic coupling by nimodipine in an animal model of cerebral small vessel disease

**DOI:** 10.1177/0271678X261417205

**Published:** 2026-02-08

**Authors:** Zhiyuan Yang, Frédéric Lange, Yiqing Xia, Ilias Tachtsidis, Kenneth J Smith

**Affiliations:** 1 Department of Neuroinflammation, UCL Queen Square Institute of Neurology, University College London, London, UK; 2 Department of Medical Physics and Biomedical Engineering, University College London, London, UK

**Keywords:** Calcium channel blocking agent, cytochrome c oxidase, non-invasive monitoring, spontaneously hypertensive stroke-prone rat, time-frequency analysis

## Abstract

Cerebral small vessel disease (cSVD) impairs the physiological mechanisms that continuously match cerebral haemodynamics to metabolic need. We have monitored this impairment non-invasively employing in-house developed broadband near-infrared spectroscopy (bNIRS) and an animal model of cSVD, the spontaneously hypertensive stroke-prone rat (SHRSP). We also assessed the vasodilating agent, nimodipine, as a potential protective treatment. Male SHRSPs were randomly allocated at 3 months of age to receive either a placebo or nimodipine diet. Both before and after 3 months on diet, the changes in the concentration of oxyhaemoglobin (HbO_2_), deoxyhaemoglobin (HHb) and oxidised cytochrome-c-oxidase (oxCCO) in the somatosensory cortex was obtained from the bNIRS recordings, and changes in blood oxygenation (HbDiff = HbO_2_ − HHb) and blood volume (HbT = HbO_2_ + HHb) were calculated. Metabolic-haemodynamic coupling was assessed by analysing the intrinsic slow wave oscillations of metabolic (oxCCO) and haemodynamic signals (HbO_2_, HHb, HbT and HbDiff) at 0.02–0.06 Hz, using wavelet coherence and semblance. Coherence and semblance were significantly (*p* < 0.001) reduced in aged SHRSPs, indicating impaired metabolic-haemodynamic coupling, but these measures were significantly (*p* < 0.001) protected by treatment with nimodipine. We reveal dysregulation of cerebral metabolic-haemodynamic coupling in SHRSP, and, importantly, demonstrate the protective effect of nimodipine, a drug suitable for clinical use in cSVD.

## Introduction

Cerebral small vessel disease (cSVD) describes pathologies affecting the small vessels of the brain, including arteriolar stiffness,^1^ deposition in the vessel wall,^2^ capillary constriction and dysfunction^3^ and vascular rarefaction.^4^ cSVD is responsible for increased risk for stroke and dementia^5^ largely due to impaired physiological mechanisms of the cerebral vasculature that constantly adjust blood flow to meet the changing metabolic demand from neurons.^6^ MRI studies have reported reduced haemodynamic responses upon visual^7,8^ or somatosensory stimulations^9,10^ in patients with cSVD and in model animals. However, the dysregulation between direct neuronal metabolism and haemodynamic response, especially in the resting state, is difficult to monitor with commonly used functional MRI techniques, but broadband near infrared spectroscopy (bNIRS) provides a solution.^11–14^ Specifically, the wavelength spectrum covered by bNIRS not only differentiates oxyhaemoglobin from deoxyhaemoglobin, but also distinguishes the oxidised cytochrome c oxidase (oxCCO) from its reduced state based on their extinction coefficients.^12,13,15^ Cytochrome c oxidase is a key component (complex IV) in the mitochondrial electron transport chain involved in aerobic oxygen metabolism, and cerebral metabolism can thus be indicated by the change in concentration of oxCCO.^11^ During neuronal activation, the oxCCO signal increases, together with HbO_2_, indicating a vascular response to increase oxygen delivery to meet the new metabolic demand, while the HHb signal decreases accordingly.^13^ Further, in the resting state, the cerebral metabolic and haemodynamic signals undergo spontaneous oscillations, termed as slow wave oscillations, with those ranging between 0.02 and 0.06 Hz considered as of neurogenic origin^16^ triggered by spontaneous electrophysiological activity.^17^ These intrinsic oscillations of HbO_2_, HHb and oxCCO and the high temporal resolution of bNIRS enable the detection of uncoupling between cerebral metabolism and haemodynamics in the resting state. Indeed, by analysing intrinsic slow wave oscillations, previous studies have confirmed an uncoupling of cerebral metabolism with haemodynamic response in patients with subarachnoid haemorrhage,^18^ and with blood pressure in newborns with hypoxic ischaemic encephalopathy.^14^

Here, we used an established animal model of cSVD, the spontaneously hypertensive and stroke prone rat (SHRSP), which mimics the epidemiological, pathological and pathophysiological features of cSVD.^19,20^ We aim to explore if metabolic-haemodynamic uncoupling can be detected from slow wave oscillations measured with bNIRS in SHRSP. Further, we have established that chronic treatment with nimodipine, a potent vasodilator of CNS vessels, restores vascular function in SHRSP^10^ and we also aim to study if the protective effect of nimodipine manifests as improved metabolic-haemodynamic coupling in the resting state.

## Materials and methods

Male SHRSPs were bred in-house and maintained in a 12-h light/dark cycle, with food and water ad libitum. The animals were randomly allocated at 3 months of age to receive either a control diet (placebo group), or a diet containing 200 mg/kg nimodipine (nimodipine group; equivalent to a daily intake of 12 mg nimodipine per kilogram body weight; Ssniff Spezialdiäten GmbH, Germany), both of which continued for 3 months. Measurements of metabolic-haemodynamic coupling were made using the bNIRS system (see below) when the animals were 3 months of age (i.e. before diet), and at 6 months of age (after 3 months on diet). The potential metabolic-haemodynamic uncoupling caused by cSVD pathology was indicated by comparing SHRSPs at 3 months of age (i.e. before the development of cerebral pathologies^19^) with animals 6 months of age. The potential therapeutic effect of nimodipine was indicated by comparing SHRSPs at 6 months old after being fed either a placebo or nimodipine diet. All experiments were performed in accordance with the UK Home Office Animals (Scientific Procedures) Act (1986), and were approved by the Ethics Committee of University College London and the UK Home Office, following the ARRIVE 2 guidelines (Animal Research: Reporting of In Vivo Experiments).

To measure metabolic and haemodynamic signals, animals were anaesthetised with 2% isoflurane in room air, maintaining body temperature at 37°C using a homeothermic heating pad (Harvard Apparatus, Cambridge, UK), and monitoring pulse oximetry, heart rate and breathing rate with a pulse oximeter (MouseOx; STARR Life Sciences, Oakmont, USA) sensor positioned on the foot. The head was stabilised using a customised stereotaxic frame, the skin overlying the somatosensory cortices 4 mm lateral and 0.2 mm rostral to bregma on both sides^21^ was shaved. The source and detector fibres of the bNIRS system were placed in close contact with the skin over the right and left somatosensory cortical regions, respectively, to account for the small size of rodent head and to obtain information from both hemispheres. A spectrometer (Ventana VIS-NIR) detected alterations in the spectrum of light across 780–900 nm emitted from a light source (HL-2000-HP) and transmitted through the somatosensory cortex between the source and the detector fibres, and relative changes in the concentration of oxyhaemoglobin (HbO_2_), deoxyhaemoglobin (HHb) and oxidised cytochrome-c oxidase (oxCCO) were calculated, as described previously.^15^

The signals of the aforementioned parameters were recorded at 0.5 Hz across a 30-min period and were processed with an automated wavelet de-noising function in MATLAB (MathWorks, MA, USA), before calculation of HbDiff (i.e. the change in oxygenation; [HbO_2_] − [HHb]) and HbT (i.e. the total blood volume; [HbO_2_] + [HHb]) to represent overall haemodynamic response. Thus, four haemodynamic parameters, namely HbO_2_, HHb, HbDiff and HbT, were used to analyse their coupling with the metabolic signal (i.e. oxCCO). The metabolic-haemodynamic coupling was determined by wavelet coherence and semblance, based on a continuous wavelet transform with the complex Morlet wavelet.^22^ Wavelet coherence represents the similarity in spectral power between the metabolic and haemodynamic signals, and varies between 0 and 1, with 1 indicating a complete dynamic synchronisation. Wavelet semblance^23^ indicates the instantaneous phase difference between the two signals, and ranges between −1 to +1, with 0 suggesting a complete absence of relationship and +1 and −1 suggesting high alignment in an in-phase and an anti-phase pattern, respectively. The average values of wavelet coherence and semblance at a frequency band between 0.02 and 0.06 Hz were calculated and compared between the three groups, namely the 3-month-old animals, the placebo group and the nimodipine group. Statistical significance was determined by individual *t*-test using MATLAB (MathWorks, MA, USA), and results indicated as mean ± SD.

## Results

**Figure 1.**
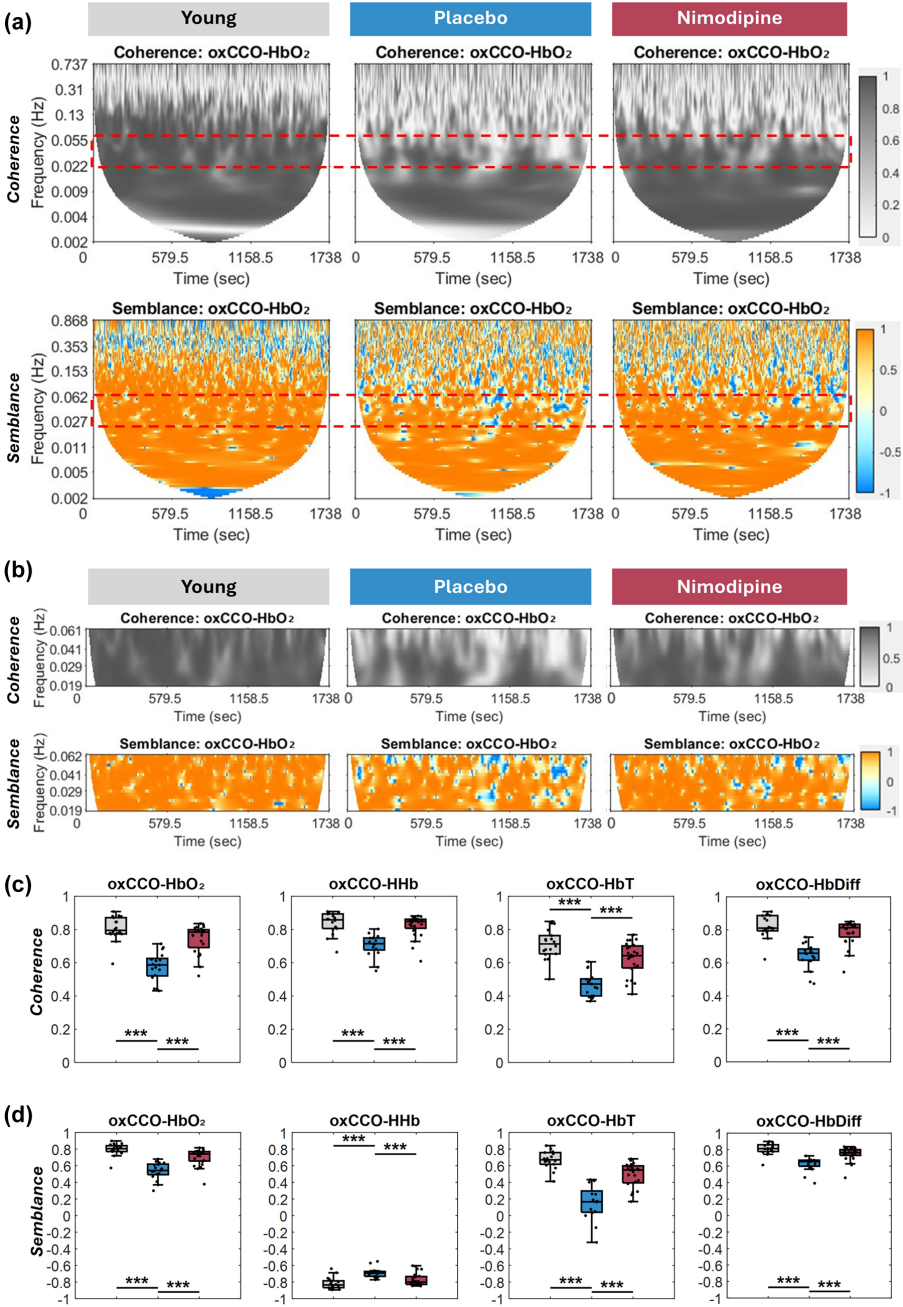
Metabolic-haemodynamic coupling is decreased in SHRSP, but protected by treatment with nimodipine. (a) Graphs illustrate coherence and semblance derived across the entire frequency range from the recorded signals in a representative animal from each of the groups (young, placebo- and nimodipine-treated), with the frequency band of interest (0.02–0.06 Hz) distinguished by the rectangle, and also separately illustrated in (b). Statistical analysis between groups showed that SHRSPs at 6 months of age (blue) showed significantly lower coherence (c) and semblance (d) between oxCCO and haemodynamic signals (HbO_2_, HHb, HbT and HbDiff) compared with young animals 3 months of age (grey). However, animals treated with nimodipine (red) showed significantly improved coherence (c) and semblance (d) in all four parameters compared with the placebo group. ***p < 0.001.

As illustrated by representative images in Figure 1(a), 3-month-old animals showed high coherence and semblance between oxCCO and HbO_2_, especially in the frequency band of interest (0.02–0.06 Hz), distinguished by the rectangle and also illustrated in Figure 1(b), while placebo-treated animals demonstrated significantly lower values in coherence (Figure 1(c)) and semblance (Figure 1(d)) between oxCCO and the four haemodynamic parameters (HbO_2_, HHb, HbDiff and HbT) compared with 3-month-old animals. Further, the animals treated with nimodipine showed significantly preserved coherence (Figure 1(c)) and semblance (Figure 1(d)) between the metabolic and haemodynamic signals compared with placebo animals of the same age. Values of metabolic-haemodynamic coupling of the three groups and statistical significance are listed in Table 1.

**Table 1.** Wavelet coherence and semblance between metabolic and haemodynamic signals in three groups of SHRSPs.

Measurements	Young (3 months, pre-treatment) (*n* = 18)	Placebo (6 months, post-treatment) (*n* = 17)	Nimodipine (6 months, post-treatment) (*n* = 24)	*p*-value	
				Young vs placebo	Placebo vs nimodipine
Coherence					
oxCCO-HbO_2_	0.80 ± 0.07	0.57 ± 0.08	0.74 ± 0.08	<0.001	<0.001
oxCCO-HHb	0.83 ± 0.06	0.70 ± 0.06	0.82 ± 0.06	<0.001	<0.001
oxCCO-HbT	0.71 ± 0.08	0.46 ± 0.06	0.62 ± 0.09	<0.001	<0.001
oxCCO-HbDiff	0.82 ± 0.06	0.64 ± 0.07	0.78 ± 0.07	<0.001	<0.001
Semblance					
oxCCO-HbO_2_	0.79 ± 0.06	0.53 ± 0.10	0.70 ± 0.09	<0.001	<0.001
oxCCO-HHb	−0.81 ± 0.06	−0.68 ± 0.05	−0.77 ± 0.07	<0.001	<0.001
oxCCO-HbT	0.67 ± 0.10	0.15 ± 0.20	0.48 ± 0.14	<0.001	<0.001
oxCCO-HbDiff	0.81 ± 0.06	0.62 ± 0.08	0.74 ± 0.08	<0.001	<0.001

HbO_2_: oxyhaemoglobin; HHb: deoxyhaemoglobin; oxCCO: oxidised cytochrome c oxidase; HbT: HbO_2_ + HHb, total haemoglobin, indicating blood volume; HbDiff: HbO_2_ − HHb, difference in oxygenation.

*p*-value: independent *t*-test. Data are presented as mean ± SD.

## Discussion

We demonstrate that metabolic-haemodynamic coupling is impaired in the SHRSP model of human cSVD, as measured by wavelet coherence and semblance detected by bNIRS. Further, we show that chronic treatment with nimodipine, an L-type calcium channel blocking agent with potent cerebrovascular activity,^24,25^ protects from the uncoupling between metabolism and haemodynamics that otherwise occurs with age.

The metabolic-haemodynamic uncoupling shown in SHRSP indicates an impairment in the ability of the diseased vasculature to match the delivery of oxygenated blood to meet the changing metabolic demand from neuronal activity, which can be detected even in the resting state. This impairment substantiates the lower haemodynamic response upon neurological stimulation (termed as neurovascular coupling, NVC) that has been extensively reported in patients with cSVD^7,8,26–30^ and in animal models.^9,10,31,32^ It is reasonable to speculate that this persistent and severe imbalance between oxygen demand and supply may initiate downstream pathological events in cSVD, including hypoxia^33–35^ and mitochondrial failure,^36^ which eventually lead to lesion formation^35^ and cognitive decline.^37^ Our study demonstrates the metabolic-haemodynamic uncoupling in prolonged disease by comparing aged SHRSP with young animals, and future studies examining normotensive animals may provide additional valuable insights.

Importantly, our findings show that the impaired metabolic-haemodynamic coupling in SHRSP can be at least partly prevented by chronic treatment with nimodipine. This protection by nimodipine observed in the resting state is an advance from our previous study which reported restored NVC in the treated animals,^10^ and from studies in other cerebral diseases.^38,39^ Observations made in the resting state address a limitation of NVC in which changes in haemodynamic response are recorded upon artificial external stimulation to activate neurons. In the case of NVC, it is not clear whether a higher haemodynamic signal is due to stronger neuronal stimulation (i.e. more surviving neurons), or a better vascular response. However, the fact that the effect of nimodipine can be observed in the resting state indicates that the protection is mainly in the vasculature, because if the vasculature remained diseased it would still show uncoupled haemodynamic signals even if tissue metabolism was enhanced by greater neuronal survival.

The observed protection of metabolic-haemodynamic coupling by nimodipine is in line with reported evidence of long-term treatment of nimodipine in preserving cognitive function (which is closely related with cerebral metabolism^40^) in patients with cerebrovascular disease^41^ and in the same model of cSVD.^10^ The mechanism underlying the protection remains unclear, not least because nimodipine has several potentially beneficial actions. The vasodilating action of the drug will reduce blood pressure (not measured in this study), and clinical studies have shown that controlling blood pressure improves vascular reactivity in patients with hypertension.^42^ However, nimodipine crosses the blood-brain barrier and it has recognised direct neuroprotective actions^43,44^ which may also be involved. Not least, nimodipine can act directly on the neurovascular unit^45^ and studies have reported a therapeutic effect of nimodipine in relaxing pericytes^46^ and preventing calcium influx into glial cells and neurons.^47,48^ Thus, although the exact mechanism(s) responsible for the protective effect of nimodipine in our study remains to be determined, the results indicate a novel therapeutic strategy for neuroprotection in cSVD, achieved in association with the preservation of mechanisms that dynamically adjust the blood flow to meet the metabolic need.

The application of bNIRS in measuring metabolic-haemodynamic coupling in the resting state, as illustrated in our study, overcomes limitations faced by common methods using functional MRI, namely the complexity of the procedure and variation caused by stimulation.^49^ Indeed, bNIRS systems boast convenience due to their portability and low-cost, and proven ability to be useful in situations difficult to cover by MRI.^14^ Further, signals obtained from functional MRI indicate changes in haemodynamics, rather than actual neuronal activity or subsequent metabolism.^50^ On the other hand, bNIRS directly measures the change in concentration of oxCCO, and thus the coupling between metabolism and haemodynamics can directly be performed with bNIRS. However, future studies are required to validate the clinical importance of metabolic-haemodynamic coupling measured from slow wave oscillations using bNIRS, and it is likely that oscillations at other frequencies, or signals obtained from other brain regions, might be a better biomarker in other neurological diseases.^16^

In conclusion, we reveal that imbalance between oxygen supply and demand in SHRSP can be indicated by measurement of metabolic-haemodynamic coupling using slow wave oscillations of bNIRS signals, preparing this protocol for future clinical use. Further, we show that nimodipine protects metabolic-haemodynamic coupling, suggesting a therapeutic value in cSVD.

## References

[1] Banerjee G Carare R Cordonnier C , et al. The increasing impact of cerebral amyloid angiopathy: essential new insights for clinical practice. J Neurol Neurosurg Psychiatry 2017; 88(11): 982–994. 28844070 10.1136/jnnp-2016-314697 PMC5740546

[2] Chabriat H Joutel A Tournier-Lasserve E , et al. CADASIL: yesterday, today, tomorrow. Eur J Neurol 2020; 27(8): 1588–1595. 32348626 10.1111/ene.14293

[3] Ostergaard L Jespersen SN Engedahl T , et al. Capillary dysfunction: its detection and causative role in dementias and stroke. Curr Neurol Neurosci Rep 2015; 15(6): 37. 25956993 10.1007/s11910-015-0557-x PMC4441906

[4] Moody DM Thore CR Anstrom JA , et al. Quantification of afferent vessels shows reduced brain vascular density in subjects with leukoaraiosis. Radiology 2004; 233(3): 883–890. 15564412 10.1148/radiol.2333020981

[5] Wardlaw JM Smith EE Biessels GJ , et al. Neuroimaging standards for research into small vessel disease and its contribution to ageing and neurodegeneration. Lancet Neurol 2013; 12(8): 822–838. 23867200 10.1016/S1474-4422(13)70124-8 PMC3714437

[6] Iadecola C. The pathobiology of vascular dementia. Neuron 2013; 80(4): 844–866. 24267647 10.1016/j.neuron.2013.10.008 PMC3842016

[7] Switzer AR Cheema I McCreary CR , et al. Cerebrovascular reactivity in cerebral amyloid angiopathy, Alzheimer disease, and mild cognitive impairment. Neurology 2020; 95(10):E1333-E40. 10.1212/WNL.0000000000010201 PMC7538216 32641520

[8] van Dijk SE Drenth N Hafkemeijer A , et al. Neurovascular decoupling is associated with lobar intracerebral hemorrhages and white matter hyperintensities. J Am Heart Assoc 2025; 14(4): e038819. 10.1161/JAHA.124.038819 PMC12074756 39950450

[9] Capone C Cognat E Ghezali L , et al. Reducing Timp3 or vitronectin ameliorates disease manifestations in CADASIL mice. Ann Neurol 2016; 79(3): 387–403. 26648042 10.1002/ana.24573 PMC5359978

[10] Yang Z Lange F Xia Y , et al. Nimodipine protects vascular and cognitive function in an animal model of cerebral small vessel disease. Stroke 2024; 55(7): 1914–1922. 38860370 10.1161/STROKEAHA.124.047154 PMC11251505

[11] Bale G Elwell CE Tachtsidis I. From Jobsis to the present day: a review of clinical near-infrared spectroscopy measurements of cerebral cytochrome-c-oxidase. J Biomed Opt 2016; 21(9): 091307. 10.1117/1.JBO.21.9.091307 27170072

[12] Tisdall MM Tachtsidis I Leung TS , et al. Near-infrared spectroscopic quantification of changes in the concentration of oxidized cytochrome c oxidase in the healthy human brain during hypoxemia. J Biomed Opt 2007; 12(2): 024002. 10.1117/1.2718541 17477717

[13] Pinti P Siddiqui MF Levy AD , et al. An analysis framework for the integration of broadband NIRS and EEG to assess neurovascular and neurometabolic coupling. Sci Rep 2021; 11(1): 3977. 33597576 10.1038/s41598-021-83420-9 PMC7889942

[14] Mitra S Bale G Highton D , et al. Pressure passivity of cerebral mitochondrial metabolism is associated with poor outcome following perinatal hypoxic ischemic brain injury. J Cereb Blood Flow Metab 2019; 39(1): 118–130. 28949271 10.1177/0271678X17733639 PMC6311664

[15] Kaynezhad P Mitra S Bale G , et al. Quantification of the severity of hypoxic-ischemic brain injury in a neonatal preclinical model using measurements of cytochrome-c-oxidase from a miniature broadband-near-infrared spectroscopy system. Neurophotonics 2019; 6(4): 045009. 31737744 10.1117/1.NPh.6.4.045009 PMC6855218

[16] Shahdadian S Wang X Kang S , et al. Site-specific effects of 800- and 850-nm forehead transcranial photobiomodulation on prefrontal bilateral connectivity and unilateral coupling in young adults. Neurophotonics 2023; 10(2): 025012. 10.1117/1.NPh.10.2.025012 PMC10240350 37284247

[17] Zuo XN Di Martino A Kelly C , et al. The oscillating brain: complex and reliable. Neuroimage 2010; 49(2): 1432–1445. 19782143 10.1016/j.neuroimage.2009.09.037 PMC2856476

[18] Highton D Ghosh A Tachtsidis I , et al. Analysis of slow wave oscillations in cerebral haemodynamics and metabolism following subarachnoid haemorrhage. Adv Exp Med Biol 2014; 812: 195–201. 24729233 10.1007/978-1-4939-0620-8_26 PMC4429250

[19] Bailey EL Smith C Sudlow CL , et al. Is the spontaneously hypertensive stroke prone rat a pertinent model of sub cortical ischemic stroke? A systematic review. Int J Stroke 2011; 6(5): 434–444. 21951409 10.1111/j.1747-4949.2011.00659.x

[20] Hainsworth AH Brittain JF Khatun H. Pre-clinical models of human cerebral small vessel disease: utility for clinical application. J Neurol Sci 2012; 322(1–2): 237–240. 22698483 10.1016/j.jns.2012.05.046

[21] Chapin JK Lin CS. Mapping the body representation in the SI cortex of anesthetized and awake rats. J Comp Neurol 1984; 229(2): 199–213. 6438190 10.1002/cne.902290206

[22] Torrence C Compo GP. A practical guide to wavelet analysis. Bull Am Meteorol Soc 1998; 79(1): 61–78.

[23] Cooper GRJ Cowan DR . Comparing time series using wavelet-based semblance analysis. Comput Geosci 2008; 34(2): 95–102.

[24] Kazda S Towart R. Nimodipine: a new calcium antagonistic drug with a preferential cerebrovascular action. Acta Neurochir (Wien) 1982; 63(1–4): 259–265. 7102417 10.1007/BF01728880

[25] Tomassoni D Lanari A Silvestrelli G , et al. Nimodipine and its use in cerebrovascular disease: evidence from recent preclinical and controlled clinical studies. Clin Exp Hypertens 2008; 30(8): 744–766. 19021025 10.1080/10641960802580232

[26] Yang S Webb AJS . Associations between neurovascular coupling and cerebral small vessel disease: a systematic review and meta-analysis. Eur Stroke J 2023; 8(4): 895–903. 37697725 10.1177/23969873231196981 PMC10683738

[27] van Dijk SE van der Grond J Lak J , et al. Longitudinal progression of magnetic resonance imaging markers and cognition in Dutch-type hereditary cerebral amyloid angiopathy. Stroke 2022; 53(6): 2006–2015. 35360926 10.1161/STROKEAHA.121.035826 PMC9126261

[28] van den Brink H Kopczak A Arts T , et al. CADASIL affects multiple aspects of cerebral small vessel function on 7T-MRI. Ann Neurol 2023; 93(1): 29–39. 36222455 10.1002/ana.26527

[29] Jokumsen-Cabral A Aires A Ferreira S , et al. Primary involvement of neurovascular coupling in cerebral autosomal-dominant arteriopathy with subcortical infarcts and leukoencephalopathy. J Neurol 2019; 266(7): 1782–1788. 31028544 10.1007/s00415-019-09331-y

[30] Huneau C Houot M Joutel A , et al. Altered dynamics of neurovascular coupling in CADASIL. Ann Clin Transl Neurol 2018; 5(7): 788–802. 30009197 10.1002/acn3.574 PMC6043774

[31] Calcinaghi N Wyss MT Jolivet R , et al. Multimodal imaging in rats reveals impaired neurovascular coupling in sustained hypertension. Stroke 2013; 44(7): 1957–1964. 23735955 10.1161/STROKEAHA.111.000185

[32] Joutel A Monet-Lepretre M Gosele C , et al. Cerebrovascular dysfunction and microcirculation rarefaction precede white matter lesions in a mouse genetic model of cerebral ischemic small vessel disease. J Clin Invest 2010; 120(2): 433–445. 20071773 10.1172/JCI39733 PMC2810078

[33] Giacalone G Zanoletti M Re R , et al. Time-domain near-infrared spectroscopy in subjects with asymptomatic cerebral small vessel disease. Appl Sci (Basel) 2021; 11(5): 2407.

[34] Lu YK Zhang C Lu XC , et al. Impact of atherosclerotic disease on cerebral microvasculature and tissue oxygenation in awake LDLR-/-hApoB+/+ transgenic mice. Neurophotonics 2019; 6(4): 045003. 10.1117/1.NPh.6.4.045003 PMC6811703 31673566

[35] Ling C Zhang ZH Wu Y , et al. Reduced venous oxygen saturation associates with increased dependence of patients with cerebral autosomal dominant arteriopathy with subcortical infarcts and leukoencephalopathy: a 7.0-T magnetic resonance imaging study. Stroke 2019; 50(11): 3128–3134. 31514698 10.1161/STROKEAHA.119.026376

[36] Denver P D’Adamo H Hu S , et al. A novel model of mixed vascular dementia incorporating hypertension in a rat model of Alzheimer’s disease. Front Physiol 2019; 10: 1269. 31708792 10.3389/fphys.2019.01269 PMC6821690

[37] Wang S Yuan J Guo X , et al. Neurochemical correlates of cognitive dysfunction in patients with leukoaraiosis: a proton magnetic resonance spectroscopy study. Neurol Res 2012; 34(10): 989–997. 23146302 10.1179/1743132812Y.0000000104

[38] Menyhart A Balint AR Kozak P , et al. Nimodipine accelerates the restoration of functional hyperemia during spreading oligemia. J Neurochem 2024; 168(5): 888–898. 36810711 10.1111/jnc.15792

[39] Szabo I Toth OM Torok Z , et al. The impact of dihydropyridine derivatives on the cerebral blood flow response to somatosensory stimulation and spreading depolarization. Br J Pharmacol 2019; 176(9): 1222–1234. 30737967 10.1111/bph.14611 PMC6468258

[40] Mori S Kato M Fujishima M. Impaired maze learning and cerebral glucose utilization in aged hypertensive rats. Hypertension 1995; 25(4 Pt 1): 545–553. 7721396 10.1161/01.hyp.25.4.545

[41] Pantoni L del Ser T Soglian AG , et al. Efficacy and safety of nimodipine in subcortical vascular dementia: a randomized placebo-controlled trial. Stroke 2005; 36(3): 619–624. 15692125 10.1161/01.STR.0000155686.73908.3e

[42] Webb AJS . Effects of vasodilating medications on cerebral haemodynamics in health and disease: systematic review and meta-analysis. J Hypertens 2019; 37(6): 1119–1125. 30540658 10.1097/HJH.0000000000002033 PMC6513078

[43] Hohmann U Ghadban C Hohmann T , et al. Nimodipine exerts time-dependent neuroprotective effect after excitotoxical damage in organotypic slice cultures. Int J Mol Sci 2022; 23(6): 3331. 35328753 10.3390/ijms23063331 PMC8954806

[44] Frank R Szarvas PA Pesti I , et al. Nimodipine inhibits spreading depolarization, ischemic injury, and neuroinflammation in mouse live brain slice preparations. Eur J Pharmacol 2024; 977: 176718. 38849040 10.1016/j.ejphar.2024.176718

[45] Attwell D Buchan AM Charpak S , et al. Glial and neuronal control of brain blood flow. Nature 2010; 468(7321): 232–243. 21068832 10.1038/nature09613 PMC3206737

[46] Korte N Barkaway A Wells J , et al. Inhibiting Ca(2+) channels in Alzheimer’s disease model mice relaxes pericytes, improves cerebral blood flow and reduces immune cell stalling and hypoxia. Nat Neurosci 2024; 27(11): 2086–2100. 39294491 10.1038/s41593-024-01753-w PMC11537984

[47] Leisz S Simmermacher S Prell J , et al. Nimodipine-dependent protection of schwann cells, astrocytes and neuronal cells from osmotic, oxidative and heat stress is associated with the activation of AKT and CREB. Int J Mol Sci 2019; 20(18): 4578. 31527507 10.3390/ijms20184578 PMC6770698

[48] Schampel A Volovitch O Koeniger T , et al. Nimodipine fosters remyelination in a mouse model of multiple sclerosis and induces microglia-specific apoptosis. Proc Natl Acad Sci U S A 2017; 114(16): E3295–E3304. 10.1073/pnas.1620052114 PMC5402421 28381594

[49] Specht K. Current challenges in translational and clinical fMRI and future directions. Front Psychiatry 2019; 10: 924. 31969840 10.3389/fpsyt.2019.00924 PMC6960120

[50] Christen T Bolar DS Zaharchuk G. Imaging brain oxygenation with MRI using blood oxygenation approaches: methods, validation, and clinical applications. AJNR Am J Neuroradiol 2013; 34(6): 1113–1123. 22859287 10.3174/ajnr.A3070 PMC7964577

